# Human Primary Olfactory Amygdala Subregions Form Distinct Functional Networks, Suggesting Distinct Olfactory Functions

**DOI:** 10.3389/fnsys.2021.752320

**Published:** 2021-12-09

**Authors:** Torben Noto, Guangyu Zhou, Qiaohan Yang, Gregory Lane, Christina Zelano

**Affiliations:** Department of Neurology, Feinberg School of Medicine, Northwestern University, Chicago, IL, United States

**Keywords:** olfaction, amygdala, medial amygdala, cortical amygdala, periamygdaloid complex, fMRI, resting connectivity

## Abstract

Three subregions of the amygdala receive monosynaptic projections from the olfactory bulb, making them part of the primary olfactory cortex. These primary olfactory areas are located at the anterior-medial aspect of the amygdala and include the medial amygdala (MeA), cortical amygdala (CoA), and the periamygdaloid complex (PAC). The vast majority of research on the amygdala has focused on the larger basolateral and basomedial subregions, which are known to be involved in implicit learning, threat responses, and emotion. Fewer studies have focused on the MeA, CoA, and PAC, with most conducted in rodents. Therefore, our understanding of the functions of these amygdala subregions is limited, particularly in humans. Here, we first conducted a review of existing literature on the MeA, CoA, and PAC. We then used resting-state fMRI and unbiased k-means clustering techniques to show that the anatomical boundaries of human MeA, CoA, and PAC accurately parcellate based on their whole-brain resting connectivity patterns alone, suggesting that their functional networks are distinct, relative both to each other and to the amygdala subregions that do not receive input from the olfactory bulb. Finally, considering that distinct functional networks are suggestive of distinct functions, we examined the whole-brain resting network of each subregion and speculated on potential roles that each region may play in olfactory processing. Based on these analyses, we speculate that the MeA could potentially be involved in the generation of rapid motor responses to olfactory stimuli (including fight/flight), particularly in approach/avoid contexts. The CoA could potentially be involved in olfactory-related reward processing, including learning and memory of approach/avoid responses. The PAC could potentially be involved in the multisensory integration of olfactory information with other sensory systems. These speculations can be used to form the basis of future studies aimed at clarifying the olfactory functions of these under-studied primary olfactory areas.

## Introduction

**Figure 1:**
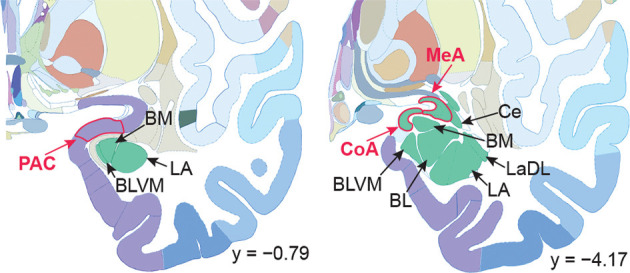
Human brain atlas with subregions of the amygdala labeled. Subregions that receive olfactory bulb input are labeled in red; others are labeled in black. Olfactory subregions include the medial amygdala (MeA), cortical amygdala (CoA), and the periamygdaloid complex (PAC). Other amygdala subregions listed for reference: the lateral amygdala (LA), ventromedial part of the basolateral amygdala (BLVM), basomedial amygdala (BM), central amygdala (Ce), and the dorsolateral part of the lateral amygdala (LaDL) (adapted from Mai et al., 2015).

After being sampled from the air by olfactory sensory neurons in the nose and synapsing through olfactory bulb glomeruli, olfactory stimuli undergo parallel processing in the brain, through at least six cortical regions, all of which receive direct, monosynaptic projections from the olfactory bulb (Carmichael et al., 1994; Lane et al., 2020). These regions, which include parts of the amygdala, comprise the primary olfactory cortex (Price, 1990, 2009; Wilson and Sullivan, 2003; Illig and Wilson, 2009 Gottfried, 2010; Mainland et al., 2014; Vaughan and Jackson, 2014; Ennis et al., 2015; Porada et al., 2019). Roughly a third of the neurons in the human primary olfactory cortex are in the amygdala (Allison, 1954), located within three subregions: the medial amygdala (MeA), the cortical amygdala (CoA), and the periamygdaloid complex (PAC) (Figure 1) (Allison, 1954; Nieuwenhuys et al., 2008; Marino et al., 2016; Weiss et al., 2021). These three amygdala subregions are poorly understood in humans. In order to explore these subregions in the human brain, this manuscript has been divided into two sections. In the first section, we reviewed the existing literature on the MeA, CoA, and PAC, comprised mostly of rodent work. In the second section, we used resting-state fMRI and unbiased k-means clustering techniques to show that the anatomical boundaries of human MeA, CoA, and PAC can be accurately parcellated based on their whole-brain resting connectivity patterns alone, suggesting that their functional networks are distinct, relative both to each other and to the amygdala subregions that do not receive input from the olfactory bulb. Further, considering that distinct functional networks are suggestive of distinct functions, we examined the whole-brain resting network of each subregion and speculated on potential specific roles that each region may play in olfactory processing.

In order to make this manuscript easier to read, we use the shorthand “olfactory amygdala” to refer collectively to the MeA, CoA, and PAC. The use of this shorthand is intended to minimize the need to list all three regions every time they are mentioned, and to reflect the fact that these are the only regions of the human amygdala that receive direct bulbar input. However, this term is not intended to imply that these regions are exclusively olfactory: in fact, they likely perform functions beyond olfaction, which remain unknown. We want to emphasize that while we focus our interpretations and framing on olfactory-guided behaviors, our findings do not show that the functions of these regions are olfactory in nature. The networks we identify may provide insight for future studies into the role of these areas in both olfactory and non-olfactory functions.

## Section 1: Literature Review of The MeA, CoA, and PAC

The amygdala consists of a collection of subregions, located in the anterior-medial temporal lobes. These subregions are distinct from each other, characterized by different cell types and connectivity (Swanson and Petrovich, 1998; Benarroch, 2015). Circuits through the amygdala are involved in a wide range of cognitive processes including face perception, implicit learning, and threat responses (Ressler, 2010; Benarroch, 2015). Much progress has been made in understanding some subregions and their corresponding behavioral circuitry (i.e., the basomedial nucleus and fear learning; Adhikari et al., 2015). However, the functional roles of some subregions remain unclear, and in particular, those which receive monosynaptic projections from the olfactory bulb are under-studied.

The anatomical organization of olfactory input to the amygdala is phylogenetically variable, suiting the specific needs of different species (Ubeda-Bañon, 2011; Abellán et al., 2013). In rodents, much of the olfactory input to the amygdala comes from the accessory olfactory system, which humans and other old-world primates lack (McDonald, 1998; Ubeda-Bañon, 2011). The rodent accessory olfactory bulb includes direct projections to the MeA and CoA (McDonald, 1998). The rodent main olfactory bulb also sends direct projections to amygdalar subregions, including the CoA (Sosulski et al., 2011), PAC (McDonald, 1998), and to a lesser extent, the MeA (Keshavarzi et al., 2015; Guthman and Vera, 2016). Both the main and accessory olfactory systems participate in chemosensory-guided social and pheromonal behaviors in rodents (Pardo-Bellver et al., 2017). In humans, the olfactory bulb projects directly to the MeA, CoA, and PAC (Crosby and Humphrey, 1941; Allison, 1954; Pereira et al., 2005). This organization of human olfactory bulb input to the amygdala is distinct from other primates including macaques, where the main olfactory bulb innervates only the PAC and CoA (Turner et al., 1978; Carmichael et al., 1994). While substantial inter-species overlap in olfactory connectivity to amygdalar subregions suggests some preservation of anatomical and functional organization, inter-species differences highlight the need for direct experimental evidence in humans (Abellán et al., 2013; Lane et al., 2020).

### The Medial Amygdala

The human MeA is situated anterior-medial to the central nucleus in the anterior-dorsal part of the amygdala (Sorvari et al., 1996; Schumann and Amaral, 2005), not to be confused with the basomedial amygdala, or the medial aspect of the amygdala (Figure 1). Most of our knowledge of the MeA comes from rodent work, which has shown that it is involved in a wide range of social behaviors (Lehman et al., 1980; Haller, 2018), and is a major constituent of the accessory olfactory system, receiving the bulk of monosynaptic projections from the accessory olfactory bulb (Mohedano-Moriano et al., 2007; Pro-Sistiaga et al., 2007). Within the accessory olfactory system, the MeA plays an important role in processing pheromonal signals and differentiating olfactory social cues including those that carry meaning about sex, age, and danger status (Bergan et al., 2014; Li et al., 2017; Yao et al., 2017; Lee et al., 2021). Social behaviors in rodents are strongly impacted by the main olfactory system (Keshavarzi et al., 2015; Pardo-Bellver et al., 2017), so it is likely that the MeA is involved in the processing of social cues that are encountered through the main olfactory system as well.

In rodents, the MeA is a multisensory area that receives cortically-processed sensory input from visual and auditory modalities (Mosher et al., 2010). The MeA is also involved in generating socially-guided behavioral outputs, including expression of aggression (Kemble et al., 1984; Blanchard and Takahashi, 1988; Newman, 1999; Veening et al., 2005; Lin et al., 2011; Hong et al., 2014; Padilla et al., 2016; Miller et al., 2019; Nordman and Li, 2020), mating behaviors (Rajendren and Moss, 1993; Kondo and Arai, 1995; Lin et al., 2011; DiBenedictis et al., 2012; Ishii et al., 2017), parenting behaviors (Fleming et al., 1980; Numan et al., 1993; Sheehan et al., 2001; Tachikawa et al., 2013; Isogai et al., 2018; Chen et al., 2019; Trouillet et al., 2019), social recognition memory (Ferguson et al., 2001; Gur et al., 2014; Shemesh et al., 2016), self-grooming (Hong et al., 2014), and interspecies defensive behaviors (Choi et al., 2005; Ishii et al., 2017; Li et al., 2017; Miller et al., 2019).

The MeA also plays a critical role in rodent approach-avoidance conflict behavior, both olfactory and non-olfactory mediated. For example, excitotoxic lesioning of the MeA reduces defensive behavior in rats during exposure to a live cat and increases exploratory locomotion (Martinez et al., 2011). Exposure to innate threat stimuli, such as predator odorants and intruder conspecifics, induces Fos expression in the MeA (Kollack-Walker et al., 1999), and distinct subpopulations of MeA neurons have opposing effects on investigation or avoidance of threatening stimuli (Miller et al., 2019). Interestingly, defensive responses are state-dependent, adapting to the fed state of an animal, and evidence suggests that these adaptations specifically involve neurons in the MeA (Padilla et al., 2016). In approach–avoidance conflict, the exploratory drive is essential to maximize an animal’s ability to thrive, whereas avoidance is essential for survival (Elliot, 2006). Findings from the aforementioned studies combine to suggest a critical role for the MeA in mediating this conflict.

Considering that the MeA is a central part of the rodent accessory olfactory system—which humans lack—the role of the MeA in humans is particularly intriguing. Non-human primate work suggests that the MeA’s involvement in social processing is conserved, showing that MeA neurons are responsive to socially important information such as facial expressions, facial identities, pair bonding, and jealousy (Leonard et al., 1985; Brothers et al., 1990; Gothard et al., 2007; Hoffman et al., 2007). Few human studies have specifically delineated the MeA and analyzed signals from it, though it may be involved in perception and processing of emotional faces (Gamer et al., 2010). Large lesions of the human amygdala that include the MeA result in emotional processing deficits, whereas lesions that spare MeA do not (Adolphs et al., 2002; Becker et al., 2012). A combined functional Magnetic Resonance Imaging (fMRI) and Positron Emission Tomography (PET) study found that connectivity between a medial portion of the human amygdala and prefrontal limbic brain regions correlated with dopamine increases in that same network when mothers interacted with their infants (Atzil et al., 2017). However, the medial portion of the amygdala used in that study was based on functional parcellations and likely corresponded to the basomedial amygdala rather than the MeA (Bickart et al., 2012). Other research on the human MeA has implicated it as part of the default mode network (Bickart et al., 2014) and it may be prone to aging and dementia-related cell loss (Herzog and Kemper, 1980; Aghamohammadi-Sereshki et al., 2019). The role of the MeA in human olfaction is virtually unexplored.

Despite this lack of research, the fact that the human olfactory bulb projects monosynaptically to the MeA (Allison, 1954) implicates this subregion in a significant olfactory role which remains to be disambiguated. Odors trigger innate responses in humans (Yeshurun and Sobel, 2010), and humans engage in olfactory-guided social behaviors (Classen, 1992; Ober et al., 1997; Wysocki and Preti, 2004; Wyart et al., 2007; Samuelsen and Meredith, 2009; de Groot et al., 2012; Frumin et al., 2015), despite the lack of an accessory olfactory system (Mast and Samuelsen, 2009; Savic et al., 2009). The neural bases of these behaviors have yet to be identified. The MeA is well-situated to process these behaviors in humans.

### The Cortical Amygdala

The human CoA is situated medial and posterior-medial to the MeA (Figure 1). In rodents, single neurons in the CoA receive input from both the main olfactory bulb and the accessory olfactory bulb (Licht and Meredith, 1987). The CoA is thought to play a role in generating innate, odor-driven behaviors, though it is likely also involved in the generation of learned olfactory responses. Additional research is needed to clarify its olfactory function. In contrast to other parts of the primary olfactory cortex, projections from the main olfactory bulb to the CoA maintain some topographical organization (Miyamichi et al., 2011; Sosulski et al., 2011). This indicates that the organization of glomeruli in the olfactory bulb is preserved in the CoA but not in other primary olfactory areas like the piriform cortex, where neurons receive input from glomeruli distributed homogenously across the olfactory bulb. The preservation of glomerular topography in the CoA is consistent with a role for this region in innate olfactory behaviors, as they are likely to be facilitated by a network with minimal layers of abstraction compared to non-stimulus-specific, learned olfactory responses. Neurons in the CoA drive innate approach/avoid behaviors in response to odors that activate the main olfactory bulb. In line with this, CoA neurons can be optogenetically controlled to trigger specific odor-guided behaviors (Root et al., 2014). Despite differences in topographical preservation between CoA and other primary olfactory areas such as the piriform cortex, odor-evoked neural ensembles in both areas are equally capable of discriminating between odors, and both exhibit similar odor tuning, reliability, and correlation structure (Iurilli and Datta, 2017).

The CoA and MeA share circuitry and have been shown to function together. In rodents, the CoA provides the MeA with much of its input from the main olfactory bulb (Keshavarzi et al., 2015), and the two regions have been shown to work together to form olfactory memories of offspring in sheep (Keller et al., 2004). The rodent CoA is highly interconnected with other amygdala areas and projects to the septum, striatum, hippocampus, and olfactory tubercle (Kevetter and Winans, 1981; Gutiérrez-Castellanos et al., 2014).

We were unable to find any human studies that specifically focused on, or reported activations in, the CoA.

### The Periamygdaloid Complex

The PAC is located anterior to the MeA and CoA (Figure 1). Relatively few studies have focused on the PAC, so much so that the region still has variable naming conventions. It is variously referred to as the periamygdaloid cortex (McDonald, 1998), periamygdalar area, anterior amygdala area (Rhone et al., 2020), and amygdalo-piriform transition area (Jolkkonen et al., 2001); some consider it part of the piriform cortex (Paxinos and Watson, 2006).

The functional role of the PAC is unclear but anatomical evidence suggests that it is an early sensory processing and integration area, as it receives direct input from both the olfactory bulb and the retina (Elliott et al., 1995). In rodents, The PAC receives input from the main olfactory bulb but not the accessory olfactory bulb, suggesting that its chemosensory function is most likely related to the main olfactory system, with less involvement in accessory olfactory processing compared to MeA and CoA (McDonald, 1998). In rodents, the PAC has notable projections to the nearby piriform cortex (Majak et al., 2004). The PAC also has bidirectional connections with the lateral amygdala (Savander et al., 1996), hippocampus (Kemppainen et al., 2002), subiculum (Krettek and Price, 1977), and striatum (Fudge et al., 2002), and is sensitive to serotonergic input (Sadikot and Parent, 1990). Lesions to PAC, CoA, and bed nucleus of the stria terminalis all reduced or eliminated attacks and signs of dominance in fights (Miczek et al., 1974), consistent with a role for PAC in social processes such as threat perception.

Research on the human PAC is extremely limited. We found a single study with a focus on the PAC in humans, identifying a role in addiction and depression (Anderson et al., 2013).

### Findings Nonspecific to Amygdala Nuclei

In many human studies, the amygdala is considered a single functional unit, and the relative contributions of MeA, CoA, PAC, and other subregions, are unspecified. This research provides evidence that the amygdala is involved in olfactory processes, but further work is needed to identify the subregions involved. Functional neuroimaging studies in humans have found increased amygdala activity in the presence of odor compared to no odor (Royet et al., 2000) and local field potentials recorded from the human amygdala show increased oscillatory activity following odor onset (Hughes and Andy, 1979; Hudry et al., 2001, 2003; Jung et al., 2006). Together, these findings support a role for the amygdala in odor perception, but the specific subnuclei that are responsive to odors are unmapped.

Human neuroimaging studies have found that activity in the amygdala correlates with participants’ reports of odor valence and intensity (Anderson et al., 2003; Winston et al., 2005; Jin et al., 2015), with no reported differences in responses between the basomedial and basolateral subdivisions (Anderson et al., 2003). This is in line with the role of the amygdala in other sensory systems (Benarroch, 2015), but whether intensity and valence of olfactory stimuli are processed by the same subregions as visual and auditory information is unknown. Evidence from patients with Urbach-Wiethe Disease, a disorder characterized by bilateral calcification of medial-anterior areas of the amygdala, suggests that damage to the medial anterior subregions of the human amygdala causes impairments to olfactory memory, facial emotion identification, and valence memory (Siebert et al., 2003). A PET study in humans found that olfactory dysfunction associated with Parkinson’s disease corresponds to an increase in acetylcholinesterase activity in the amygdala as well as in other limbic and olfactory areas (Bohnen et al., 2010). Thus, pathology in olfactory amygdala circuitry may represent a disruption in a circuit that links olfactory information with the rest of the limbic system, in addition to the piriform cortex, which may have been assumed to provide the entirety of this link in the past. These pieces of evidence link olfactory dysfunction to the amygdala, supporting a key, under-considered role for the amygdala in healthy olfactory processing.

Olfactory processing is inherently linked to respiration (Mainland and Sobel, 2006). Growing evidence suggests that activity in medial and anterior areas of the human amygdala are related to breathing. Local field potential oscillations in the amygdala increase with nasal inhalation (Heck et al., 2016; Zelano et al., 2016; Herrero et al., 2018), electrically stimulating medial and anterior areas of the human amygdala halts nasal breathing (Dlouhy et al., 2015; Lacuey et al., 2017; Nobis et al., 2018), and the timing of seizure spread to the amygdala correlates with the timing of seizure-induced apnea (Nobis et al., 2019). Furthermore, breathlessness and respiratory modulations have been associated with medial and anterior areas of the amygdala overlapping with the MeA, CoA, and PAC (Masaoka and Homma, 2001). A potential pathway by which amygdala activity may impact breathing behavior lies in the fact that the central nucleus of the amygdala projects to numerous respiratory areas of the brainstem in macaques (Price and Amaral, 1981).

In summary, these studies show that the human amygdala is involved in olfactory processing, but the specific subregions that carry out these processes have yet to be defined. Moreover, anterior-medial areas of the amygdala that receive olfactory input are involved in other processes such as memory and respiratory modulation. The fact that the amygdala is part of the primary olfactory cortex contrasts with the anatomical organization of other sensory systems, in that primary olfactory circuitry is entangled within a host of other cognitive processes in the amygdala. By investigating the resting connectivity of each olfactory subregion of the amygdala, we aim to better understand both their olfactory and extra-olfactory roles.

## Section 2: Characterizing Whole-Brain Networks of Human MeA, CoA, and PAC

Little is known about the subregions of the human amygdala that receive olfactory input: the MeA, CoA, and PAC. Here, we used resting-state fMRI techniques to describe whole-brain functional networks of these three human olfactory amygdala subregions, with two main goals. Our first goal was to determine whether whole-brain resting connectivity networks could be used to accurately parcellate the anatomical boundaries of MeA, CoA, and PAC: If true, this would suggest that their resting networks are distinct, which would imply that their functions are distinct. Our second goal was to describe the resting whole-brain connectivity patterns of each olfactory amygdala subregion.

## Materials and Methods

This data has been previously reported in Zhou et al. (2019).

### Participants

Functional resting-state data were collected from 25 healthy participants (14 female) with an average age of 25.5 (standard error: 1.2) years. All participants were right-handed, and none had a history of psychiatric, neurological, smell, taste, or respiratory disorders. This study was approved by Northwestern University’s Institutional Review Board under Protocol #STU00201746, was conducted in accordance with the Declaration of Helsinki, and all participants gave their voluntary written consent. Participants were instructed to look at a white fixation cross on a black background and to breathe in and out through their noses for 10 min while neuroimaging data were collected.

### fMRI Data Acquisition

fMRI data were collected at Northwestern University’s Center for Translational Imaging using a 3T Siemens TIM Trio scanner equipped with a 64-channel head coil (Siemens Healthcare, Erlangen, Germany). Scans were acquired using a single-shot gradient-echo planar-imaging sequence with the following parameters: repetition time (TR): 555 ms; echo time (TE): 22 ms; flip angle: 47°; MB-8 with Split-slice GRAPPA (Olman et al., 2009; Todd et al., 2016); field of view (FOV): 208 mm; voxel size: 2.0 × 2.0 × 2.0 mm^3^; 64 axial slices. The slice orientation was set to approximately 30° from the AC-PC line (Deichmann et al., 2003) to reduce the distortion and improve the signal-to-noise ratio in the primary olfactory and orbitofrontal areas (Zhou et al., 2019). A T1-weighted MPRAGE high-resolution anatomical image was acquired for each participant with the following parameters: TR: 2,300 ms; TE: 2.94 ms; FOV: 256 mm; flip angle: 9°; voxel size: 1.0 × 1.0 × 1.0 mm^3^; 176 sagittal slices.

### fMRI Data Preprocessing

MRI data were preprocessed using FSL (FMRIB Software Library^1^; RRID:SCR_002823; Smith et al., 2004; Woolrich et al., 2009; Jenkinson et al., 2012). Structural images were skull-stripped and segmented into gray matter, white matter, and cerebral spinal fluid using FSL’s BET (Smith, 2002) and FAST (Zhang et al., 2001) tools. White matter and cerebrospinal fluid images were eroded 1 voxel (FSL’s fslmaths) to avoid false rejections of gray matter voxels.

The first 10 volumes of the fMRI volumes were removed, and the resting data were motion-corrected and registered to the individual anatomical image using the brain-boundary registration method. The anatomical image of each participant was registered to the Montreal Neurological Institute (MNI) standard brain (MNI152_T1_2mm_brain) using the non-linear registration method with 12 degrees of freedom. Linear and quadratic trends were removed using Analysis of Functional NeuroImages (AFNI; RRID:SCR_005927; Cox, 1996). Nuisance variables, including six head-movement parameters, and white matter and cerebrospinal signals, were regressed out using multiple linear regression methods (FSL’s fsl_glm). Finally, images were intensity normalized, band-pass filtered (0.008–0.01 Hz, AFNI’s 3dFourier), registered to MNI space, and spatially smoothed (Gaussian kernel, sigma = 3).

### Parcellation of Amygdala Subregions With k-Means Clustering

In our first two analyses, we parcellated the amygdala into subregions using unsupervised k-means clustering on whole-brain resting connectivity. To do this, we calculated the Pearson correlation coefficient between each voxel within the mask and every other voxel in the rest of the brain. The whole-brain mask was created by thresholding FSL’s gray matter image (tissue prior image avg152T1_gray.img) at a threshold of 100. The resulting correlation matrix was Fisher z transformed and averaged over participants, resulting in a group-level correlation matrix, which was transformed back into Pearson correlation coefficients. Finally, we used standard k-means methods (Matlab’s Statistics Toolbox) to conduct the parcellation analysis, in which the correlation between the connectivity patterns of the voxels was used as the distance measure.

We used a permutation analysis to calculate the statistical significance of the parcellation accuracy. In each permutation, the labels of the anatomical subdivisions were shuffled, and the percent of olfactory subregions of the amygdala overlapping with the permuted set was calculated. We repeated this procedure 10,000 times to get a distribution of the proportion of voxels of each parcellated subdivision within each anatomical subregion. Matlab’s *normfit* function was used to perform normal distribution fitting of the permuted data and a z score of the actual proportion values was computed by subtracting the average and then dividing by the standard deviation of the distribution.

## Result 1: Olfactory Amygdala Subregions Are Dissociable from Non-Olfactory Amygdala Subregions Based on Whole-Brain Connectivity Patterns

**Figure 2:**
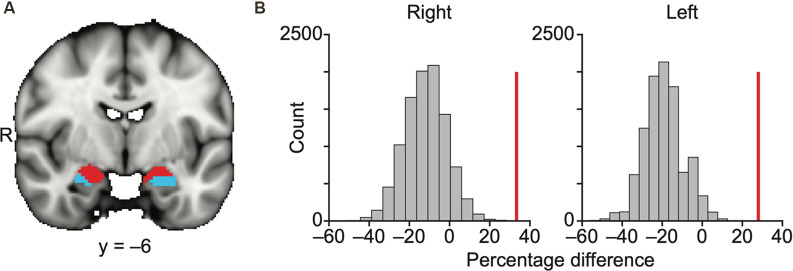
Olfactory and non-olfactory areas of the amygdala are delineated using resting state connectivity. **(A)** Results of k-means (*k* = 2) clustering of the amygdala based on resting state connectivity. Each color (red and blue) in panel **(A)** indicates one cluster. The data are shown on the FSL’s MNI152_T1_1 mm_brain. **(B)** Bar plots indicate the distribution of permuted difference of the percentage of the olfactory subregions within each parcellation. The red vertical line indicates real value. Data are shown for the right and left hemispheres separately. R, right hemisphere.

We were first interested in determining whether the amygdala subregions that receive direct bulb input (referred to here as olfactory subregions) have distinct whole-brain resting connectivity patterns relative to the amygdala subregions that do not receive direct bulb input (referred to here as non-olfactory subregions). To examine whether the olfactory and non-olfactory amygdala subregions are dissociable according to their whole-brain functional connectivity patterns, we parcellated a whole-amygdala mask into subregions based on connectivity patterns with the rest of the brain using a well-established, unbiased k-means clustering resting connectivity parcellation method (Kahnt et al., 2012; Zhou et al., 2019; “Materials and Methods” section). In order to test whether olfactory and non-olfactory subregions would emerge as distinct areas, the number of clusters was set to 2. The analysis was performed for the left and right hemispheres separately. The results showed that the combined mask was successfully parcellated into two distinct clusters in each hemisphere, each of which corresponded predominantly with either the olfactory or non-olfactory subregions (Figure 2A).

We used a permutation method to statistically quantify how accurately these two parcellated clusters corresponded to the olfactory and non-olfactory subregions (Figure 2B, “Materials and Methods” section). In each permutation, the anatomical labels of the parcellation clusters in the amygdala were randomly shuffled across voxels, and the percentage of the olfactory amygdala subregions (any voxels within MeA, CoA, or PAC) within each k-means parcellation was calculated. The difference in the percentage of the olfactory amygdala subregions within the two clusters (olfactory vs. non-olfactory) was calculated in 10,000 permutations, resulting in a null distribution and a z score of the real percentage difference. We found that the olfactory amygdala subregions were more likely to fall into the same cluster for both the right hemisphere (*z* = 4.27, *P* = 2.0e–6) and left hemisphere (*z* = 4.79, *P* = 1.7e–6; Figure 2B).

## Result 2: Olfactory Amygdala Subregions Form Distinct Whole-Brain Networks

**Figure 3:**
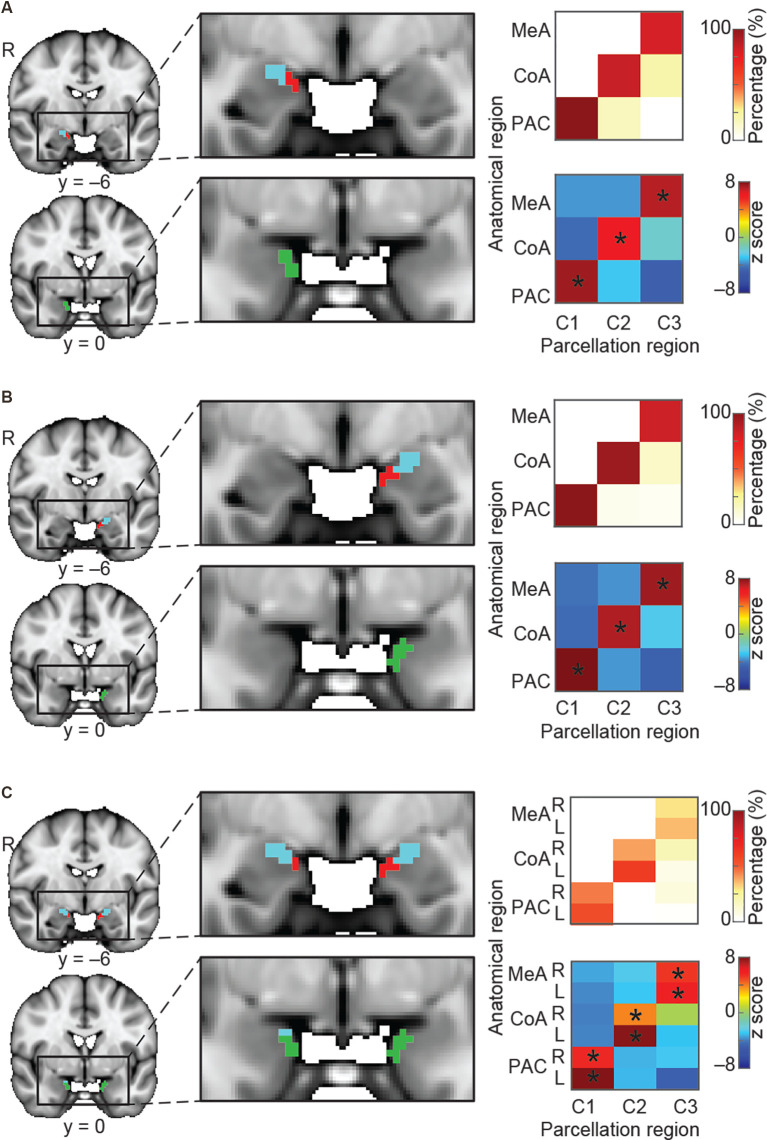
Parcellation of the olfactory amygdala subregions based on resting state connectivity. K-means (*k* = 3) clustering analysis was performed for the right hemisphere **(A)**, left hemisphere **(B)**, and combined left and right **(C)** hemispheres. Each color (red, blue, and green) in **(A–C)** indicates one cluster. Results are shown on the FSL’s MNI152_T1_1 mm_brain. Parcellation accuracy of each subregion is shown on the right. Top right: proportion of voxels from each parcellation subdivision located within each anatomical subregion. Bottom right: z score of the proportion maps. *Indicates *P* < 0.05 (Bonferroni corrected). L, left hemisphere; R, right hemisphere; MeA, medial amygdala; CoA, cortical amygdala; PAC, periamygdaloid complex.

So far, we have demonstrated that the amygdala subregions receiving direct input from the olfactory bulb have distinct whole-brain resting connectivity patterns compared to other amygdala subregions. We next tested the hypothesis that each of the three olfactory amygdala subregions also has distinct, whole-brain functional connectivity patterns relative to each other. To this end, we asked whether we could use resting whole-brain connectivity maps to accurately delineate their anatomical boundaries. To do so, we performed a k-means clustering analysis focused on the olfactory amygdala subregions. We combined regions of interest (ROIs) of the MeA, CoA, and PAC into one mask, and performed the same whole-brain connectivity-based parcellation procedure as described above. For this analysis, in order to test whether the three olfactory subregions would emerge as distinct areas, the number of clusters was set to 3, and the analysis was performed for the left and right hemispheres separately. Results showed that the *k* = 3 solution yielded three distinct clusters that corresponded accurately to the MeA, CoA, and PAC for both the right (Figure 3A) and left (Figure 3B) hemispheres. To confirm the correspondence between the anatomical delineation of the olfactory amygdala subregions in the Atlas of the Human Brain (Mai et al., 2015) and our parcellation results, we computed the proportion of voxels from each parcellation cluster located within each of the atlas-derived subdivisions, which were drawn prior to the parcellation analysis (Zhou et al., 2019). The statistical significance of this proportion was tested using a permutation method. The results indicated that, in both the left and right amygdala, each parcellated cluster corresponded to a single anatomically-defined ROI. For each parcellated subdivision, a single corresponding anatomical ROI contained significantly more voxels than other anatomical ROIs (Figures 3A,B right, minimum *z* = 6.05). This confirmed that the MeA, CoA, and PAC have distinct whole-brain functional networks.

We next asked whether the resting state networks of each subregion are similar across left and right hemispheres. To do this, we computed the same connectivity-based parcellation as described above, but using a combined mask which included both left and right amygdala. We hypothesized that if the whole-brain functional connectivity patterns of each subregion were similar across hemispheres, then we would find that the left and right portions of each subregion would cluster together. For example, we would expect that the right MeA would cluster with the left MeA rather than the right CoA, and so on. We indeed found that the bilateral amygdala mask parcellated into three subdivisions, each of which included the left and right portions of each subregion: One cluster included left and right MeA, one cluster included left and right CoA, and one cluster included left and right PAC (Figure 3C). Given that each subregion is anatomically closer to the other subregions in the same hemisphere than it is to the same subregion on the opposite hemisphere, this evidence strongly suggests that resting state networks of the left and right amygdala subregions are similar. Based on this result, whole-brain functional connectivity maps for subsequent analyses were conducted collapsed across left and right hemispheres. A validation analysis confirmed that setting k to values greater than 3 results in clusters that do not match olfactory areas, likely reflecting other amygdala networks (Supplementary Figure 1).

## Result 3: Distinct Networks of Mea, Coa, and Pac

**Figure 4:**
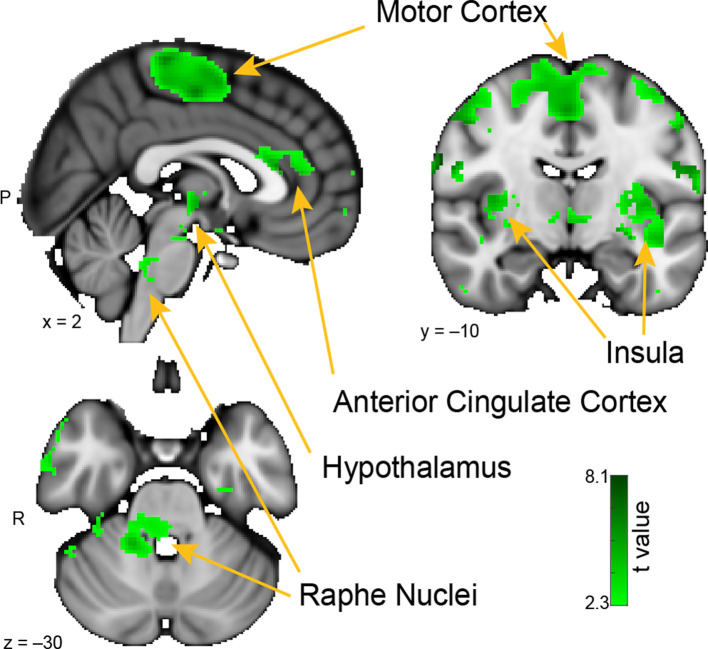
Whole-brain resting connectivity of the MeA. Regions of interest include the insula, motor cortex, anterior cingulate cortex, and raphe nuclei. The connectivity map was thresholded at threshold-free cluster enhancement corrected *P* < 0.05. Results are overlaid on FSL’s MNI152_T1_1 mm_brain.

**Figure 5:**
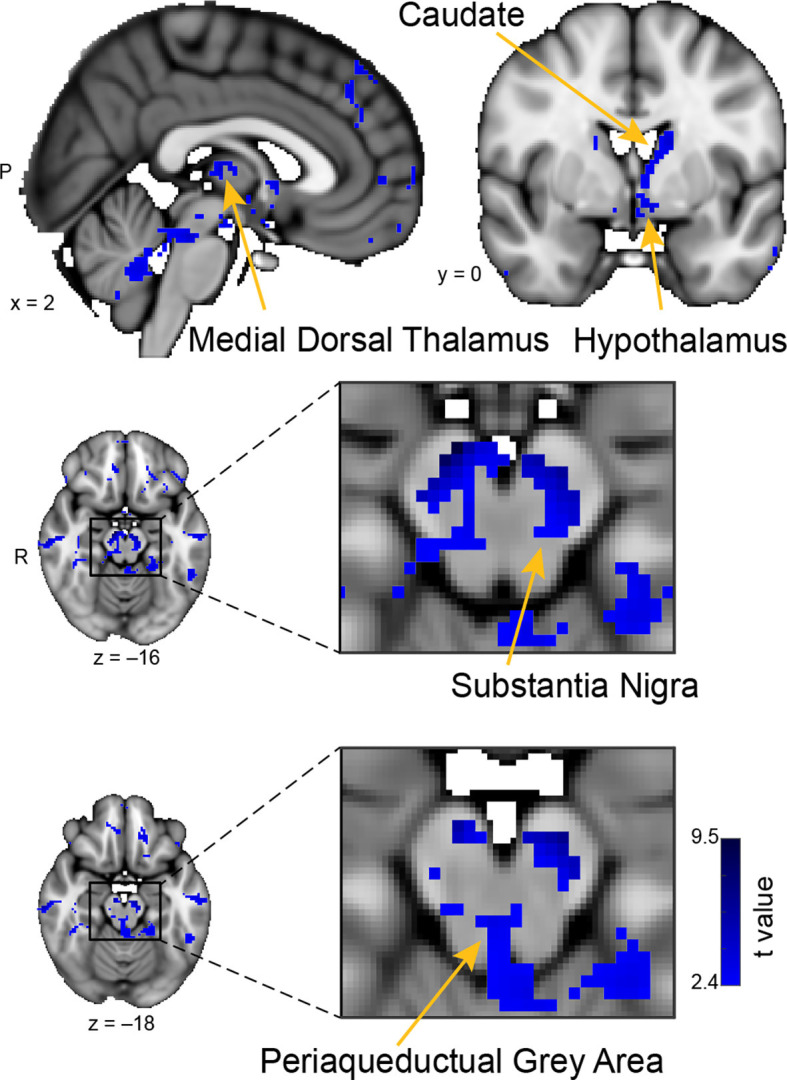
Whole-brain resting connectivity of the CoA. Regions of interest include the hypothalamus, substantia nigra, periaqueductal gray area, and medial dorsal thalamus. The connectivity map was thresholded at threshold-free cluster enhancement corrected *P* < 0.05. Results are overlaid on FSL’s MNI152_T1_1 mm_brain.

**Figure 6:**
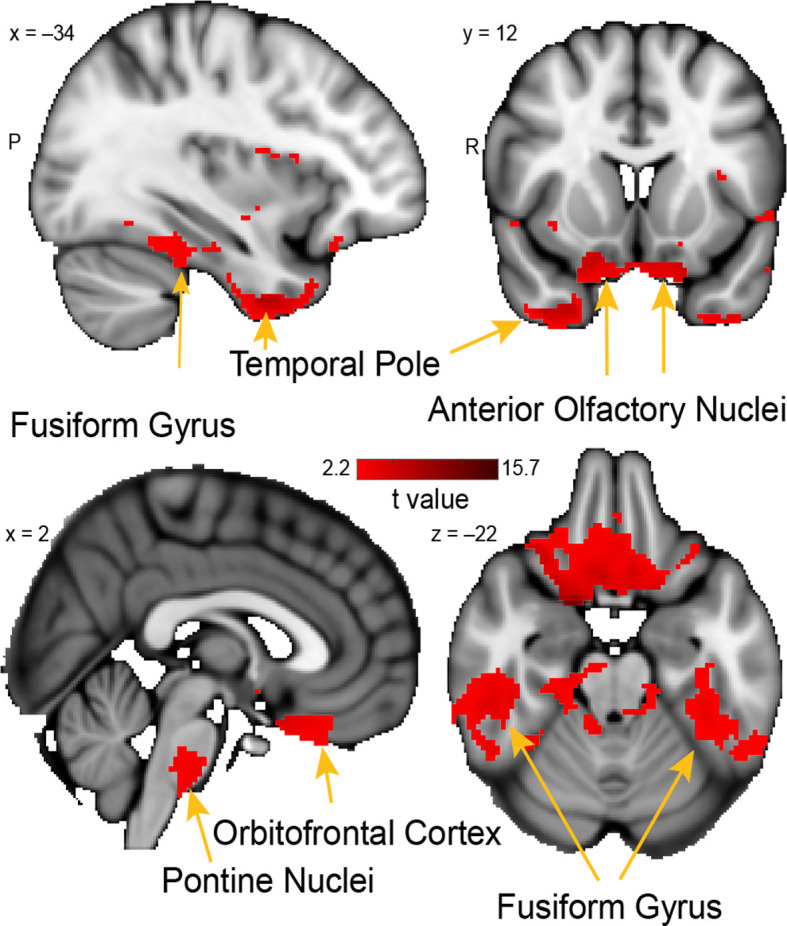
Whole-brain resting connectivity of the PAC. Regions of interest include the fusiform gyrus, anterior olfactory nucleus, pontine nucleus, and orbitofrontal cortex. The connectivity map was thresholded at threshold-free cluster enhancement corrected *P* < 0.05. Results are overlaid on FSL’s MNI152_T1_1 mm_brain.

Thus far, we have demonstrated that olfactory subregions of the amygdala have distinct resting connectivity profiles. We next sought to describe and characterize the whole-brain functional connectivity maps of each olfactory subregion. To do this, we generated whole-brain, non-overlapping maps of the voxels exhibiting functional connectivity with each amygdala subregion. Specifically, we first applied a statistical threshold to the whole-brain functional connectivity map for each subregion (threshold-free cluster enhancement (TFCE) corrected *P* < 0.05) to assign a value of 1 or 0, resulting in a binarized map for each subregion. We then further masked these maps according to whether each voxel exhibited statistically significant functional connectivity with a single subregion or with multiple subregions. This produced two categories of functional connectivity maps: one containing unique connectivity patterns for each olfactory subregion, and the other containing connectivity patterns shared by multiple olfactory subregions (see “Result 4: Combined Analysis” section). Here we describe the distinct, whole-brain resting networks that we found, exclusive to each subregion (Figures 4–6). This analysis is agnostic to whether the resting state map for each olfactory subregion reflects olfactory or non-olfactory circuits. By generating these maps, we can better design future studies that test their function directly.

### The Medial Amygdala

The brain areas that showed connectivity unique to the MeA were located in the hypothalamus, the insula, pre- and post-central gyri, and the superior temporal gyrus (Figure 4). Additional connectivity clusters were evident in the globus pallidus, putamen, caudate, and brainstem. Within the hypothalamus, the cluster of connectivity unique to the MeA was restricted to the posterior area, which is involved in the generation of the fight-or-flight response to deal with imminent threats (Shekhar and Dimicco, 1987; Falkner and Lin, 2014). Connectivity between the MeA and the insula was extensive, including clusters located in both posterior and anterior areas. Large connectivity clusters were found across premotor and motor cortices, with smaller clusters in the globus pallidus, caudate, and putamen, all areas involved in motor planning and movement. Within the brainstem, we observed a distinct cluster of significant MeA connectivity in the dorsal pons, corresponding to the raphe nuclei.

### The Cortical Amygdala

The unique whole-brain functional network of the CoA largely included areas in the midbrain and brainstem, with some additional clusters in the hippocampus, middle temporal gyrus, and septal areas (Figure 5). Within the midbrain, there was connectivity with the mediodorsal thalamus, which is involved in olfactory learning and memory (Courtiol and Wilson, 2015). There was also extensive connectivity with the substantia nigra, including ventral, medial, and lateral subdivisions, which are involved in value and salience coding (Zhang et al., 2017). Within the brainstem, connectivity was evident with the periaqueductal gray. There were also large connectivity clusters in the posterior hippocampus and the middle temporal gyrus. The middle temporal gyrus is associated with the recognition of known faces and emotional recognition (Pourtois et al., 2005). Within septal areas, there was a cluster of connectivity corresponding to the posterior parolfactory cortex.

### The Periamygdaloid Complex

The unique whole-brain functional network of connectivity with the PAC included large clusters in the anterior olfactory nucleus, brainstem, fusiform cortex, and the temporal pole (Figure 6). Additional connectivity was evident in the entorhinal cortex and orbitofrontal cortex. Connectivity between PAC and the anterior olfactory nucleus—a multifunctional cortical area, despite the name, which provides extensive ongoing feedback to the olfactory bulb (Rothermel and Wachowiak, 2014)—was extensive, covering the entire anterior-posterior axis of this primary olfactory area. In the brainstem, there was a single large cluster of significant voxels covering the pontine nuclei, which consists mainly of neurons linking the cerebral cortex to the cerebellum (Glickstein et al., 1985). There was bilateral connectivity with the fusiform face area, which is strongly responsive to faces (Kanwisher et al., 1997; Grill-Spector et al., 2004). There was also a large cluster of connectivity with the temporal pole, an area that has been associated with social and emotional processing (Olson et al., 2007).

## Result 4: Combined Analysis

**Figure 7:**
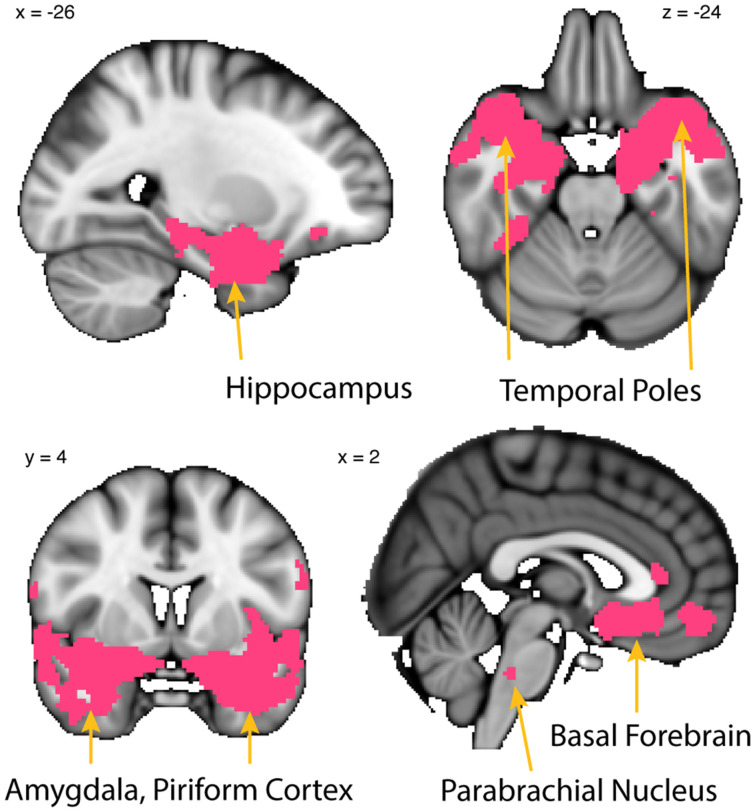
Whole-brain resting connectivity is common to MeA, CoA, and PAC. Regions of interest include the piriform cortex, entorhinal cortex, olfactory tubercle, temporal pole, basal forebrain, and other areas of the amygdala. The connectivity maps were thresholded at threshold-free cluster enhancement corrected *P* < 0.05. Results are overlaid on FSL’s MNI152_T1_1 mm_brain.

**Figure 8:**
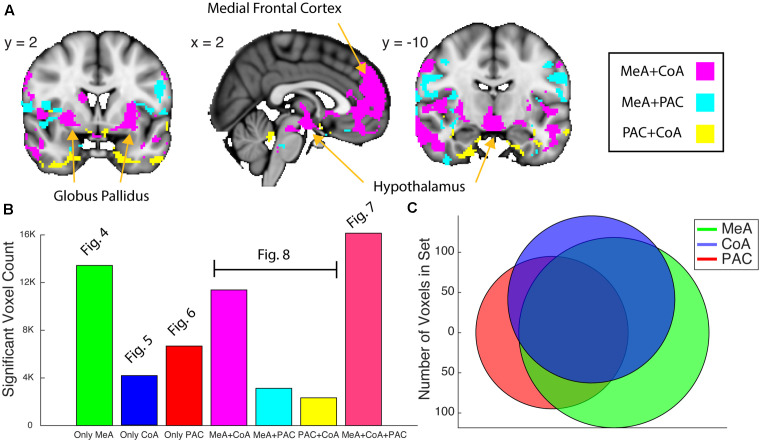
Whole-brain resting connectivity common to two of the three olfactory amygdala subregions. **(A)** Intersections of resting state networks of olfactory amygdala subregion pairs. Results are overlaid on FSL’s MNI152_T1_1 mm_brain. **(B)** Bar plot of the number of voxels that shows significant connectivity with each (Figures 4–6) and combination (panel **A** and Figure 7) of the olfactory subregions. **(C)** Venn Diagram of panel **(B)**. The connectivity maps were thresholded at threshold-free cluster enhancement corrected *P* < 0.05.

Thus far, we have shown that primary olfactory amygdala subregions can be distinguished from non-olfactory amygdala subregions (Result 1, Figure 2) and from each other (Result 2, Figure 3), based on their functional connectivity profiles. We have also mapped each subregion’s distinct connectivity profile (Result 3, Figures 4–6). We next examined the functional pathways that these subregions share in common. This analysis was motivated by the fact that amygdala subregions are highly interconnected, and that certain groups of subregions work in concert during certain cognitive tasks (Benarroch, 2015). For instance, a given functional network may rely on processing in the MeA and CoA, but not the PAC, and another may rely on processing in all three olfactory amygdala subregions. By comparing the overlapping resting state networks, we may gain insight into circuits that engage multiple olfactory amygdala subnuclei. To examine the shared networks, first, we mapped the whole-brain resting-state connectivity patterns shared by all three subregions (Figure 7), then compared sets of two subregions (Figure 8).

### Whole-Brain Connectivity Common to MeA, CoA, and PAC

To identify the network common to all three subregions, the connectivity map of each subregion was binarized at a threshold of TFCE corrected *P* < 0.05 to include only those clusters that exhibited connectivity with all three subregions. This resulted in a whole-brain connectivity map of areas that are functionally connected to all olfactory amygdala subregions (Figure 7). The MeA, CoA, and PAC all had significant resting state connectivity with the hippocampus, parahippocampal gyrus, other subregions of the amygdala, bed nucleus of the stria terminalis, the medial temporal pole, and the dorsal pons. There was also significant resting connectivity with other subregions of the primary olfactory cortex including piriform cortex, entorhinal cortex, and the olfactory tubercle, but not the anterior olfactory nucleus (which had connectivity specific to the PAC). The cluster of significant resting state connectivity in the posterior pons possibly corresponds to the parabrachial nucleus, although defining human brainstem subregions is in its infancy (Lavezzi et al., 2004).

### Whole-Brain Networks Shared by Sets of Two Olfactory Amygdala Subregions

We next examined the networks shared by each pair of subnuclei. We found that the MeA and CoA had more similar whole-brain functional connectivity networks compared to PAC, which differed substantially. To quantify this, we computed a Venn Diagram of voxel overlaps (Figure 8). Indeed, MeA and CoA had more overlapping voxels of connectivity compared to PAC with either MeA or CoA (Figure 8). We mapped the voxels showing significant resting connectivity with each set of two subregions, for each combination of subregions (PAC and MeA, PAC and CoA, and MeA and CoA). The set of voxels correlating with the PAC and MeA, but not CoA, was small, with scattered patterns of significance in the temporal lobes, orbitofrontal cortex, and the hypothalamus. The set of voxels correlating with the PAC and CoA, but not MeA, was also small, with spotted coverage in the operculum and insula. However, a larger set of voxels correlated with the MeA and CoA, but not the PAC. These areas included the medial frontal cortex, the hypothalamus, and the globus pallidus. We have included supplementary figures representing the MeA, CoA, and PAC’s whole-brain resting state networks that include overlap with other olfactory subregions (Supplementary Figure 2).

## Discussion

The goal of this study was to characterize the whole-brain resting networks of the amygdala subregions that receive direct input from the olfactory bulb in humans: MeA, CoA, and PAC. First, we reviewed the existing literature on the anatomical and functional properties of each subregion. Then, we used resting state fMRI to show that these three subregions could be accurately parcellated based on their whole-brain connectivity patterns, suggesting distinct resting networks. Finally, we described the distinct resting network of each subregion, revealing the unique set of brain areas exhibiting connectivity with each primary olfactory amygdalar subregion, as well as those areas with common connectivity. These areas of connectivity may provide an initial starting place to form hypotheses for future studies into the potential functional properties of these brain areas, which have been understudied, particularly in humans.

In considering these results, an overall picture of olfactory amygdala subregion networks begins to emerge: These three subregions receive direct projections from the olfactory bulb and make up about a third of the primary olfactory cortex, suggesting that they play a role in processing olfactory input. They participate in a shared network, though each subregion also has a distinct and more specific network. We found that all three amygdala subregions share connectivity with brain areas involved in social and emotional behavior, and with brain areas involved in autonomic functions such as respiration, heart rate, and blood pressure. This suggests that there may be a social/emotional context for the specific functions performed by each subregion, and that these parts of the amygdala could be involved in mediating changes in autonomic functions based on olfactory, social, and emotional input. Consideration of the unique connectivity of each network may allow for speculation of the unique functions of each subregion within these shared contexts.

The whole-brain network that we identified for the MeA revealed connectivity with a number of areas involved in the generation of fight or flight responses (hypothalamus, raphe nuclei) and movement (globus pallidus, caudate, motor cortex, and ventral thalamus). The whole-brain network we identified for the CoA revealed connectivity with a number of areas involved in midbrain reward circuitry (caudate, substantia nigra, and periaqueductal gray), learning (mediodorsal thalamus), and memory (posterior hippocampus). The whole-brain network that we identified for the PAC revealed connectivity with areas involved in olfactory cognition (anterior olfactory nucleus and orbitofrontal cortex) and multisensory integration (temporal pole and pontine nuclei). Based on these findings, we can speculate on potential roles for the MeA, CoA, and PAC in human olfactory processing, forming ideas that can be tested in future studies. The MeA could potentially be involved in the generation of rapid motor responses to olfactory stimuli, particularly in approach/avoid contexts. The CoA could potentially be involved in olfactory-related reward processing, including learning and memory of approach/avoid responses. The PAC could potentially be involved in the multisensory integration of olfactory information with other sensory systems.

### Validation of Distinct Resting State Networks

Clusters of voxels in the amygdala are defined purely by resting state networks, tightly aligned with the anatomical boundaries of the subregions that receive olfactory bulb input (Figure 2). This corresponds to other studies showing that the amygdala can be functionally parcellated into anterior-medial and basolateral regions (Bach et al., 2011; Bzdok et al., 2013; Bielski et al., 2021), though these studies did not distinguish olfactory from non-olfactory subregions. A similar parcellation analysis using only the voxels within subregions of the amygdala that receive olfactory bulb input in humans revealed that the clusters tightly aligned to the anatomical boundaries of each olfactory amygdala subregion (Figure 3). This held true when voxels from bilateral olfactory amygdala subregions were analyzed (Figure 3C), demonstrating that the resting connectivity of voxels within an olfactory subregion are more similar to those of the corresponding subregion on the contralateral hemisphere compared to those for ipsilateral, neighboring subregions. This evidence strongly conveys that the human MeA, CoA, and PAC have distinct whole-brain resting state networks.

### The Medial Amygdala

Based on the resting connectivity map of the MeA, which covers cortical and sub-cortical motor areas, the hypothalamus, and anterior cingulate cortex, our results are consistent with other work (Nordman and Li, 2020) supporting the idea that the MeA plays a central role in generating fight-or-flight responses. In the human brain, the MeA receives monosynaptic input from the olfactory bulb (Allison, 1954), and could potentially be involved in the generation of rapid motor responses to odors.

The resting connectivity map of the MeA included areas of the motor cortex. This corresponds with human DTI evidence (Grèzes et al., 2014), which has shown that fiber tracts originating near the MeA project throughout the motor cortex, in line with tracer findings in macaques (Morecraft et al., 2007). This amygdala-motor pathway is considered the basis of innate emotional behaviors such as smiling (Gothard, 2014) and fight or flight responses (Sagaspe et al., 2011). The MeA also had significant resting connectivity with the anterior cingulate cortex, a brain region involved in anxiety (Straube et al., 2009).

In the midbrain, we observed clusters of voxels with significant resting state connectivity in the posterior and lateral hypothalamic areas corresponding to fight or flight, social, and neuroendocrine bonding processes (Shekhar and Dimicco, 1987; Choi et al., 2005; Sivukhina and Jirikowski, 2021). The MeA showed resting connectivity with areas of the brainstem including the raphe nuclei. In macaques, the amygdala projects to numerous areas of brainstem, most of which project back reciprocally (Price and Amaral, 1981). The raphe nuclei produce most of the brain’s serotonin (Hornung, 2003), have been shown to exhibit functional connectivity with the human amygdala (Beliveau et al., 2015), and are involved in fight or flight responses (Kuwaki, 2021). In rodents, the raphe nuclei project heavily to the olfactory bulb (McLean and Shipley, 1987; Steinfeld et al., 2015), where they have been shown to modulate its output (Kapoor et al., 2016). Our finding of functional connectivity between MeA and raphe nuclei suggests that these nuclei could potentially provide a route by which MeA can modulate activity in the olfactory bulb.

The MeA also showed significant resting state connectivity throughout the insula. Nearly all subregions of the amygdala are reciprocally connected with the insula (Mufson et al., 1981) and amygdala-insula connections are thought to govern disgust, appetite, reward, and satiety (Sarinopoulos et al., 2006; Boutelle et al., 2015; Langenecker et al., 2020). Further research is needed to understand whether extensive connectivity between the MeA and the insula represents an olfactory function.

### The Cortical Amygdala

The resting connectivity map of the CoA included areas involved in reward processing, motivation, and olfactory learning and memory, suggesting that the CoA could potentially be involved in olfactory-related reward processing, including learning and memory of approach/avoid responses.

The resting state connectivity map of the CoA included the caudate and substantia nigra, both of which have been shown to exhibit connectivity with the amygdala, as components of a well-studied limbic anticipatory-reward circuit (Lee et al., 2006; Langenecker et al., 2020). The CoA also showed significant resting state connectivity with the periaqueductal gray (PAG) area, which is involved in respiratory control and motivated behaviors (Motta et al., 2017). PAG is also involved in defensive behaviors (Tovote et al., 2016), and especially respiratory defensive behaviors like breath-holding (Faull et al., 2015, 2016). In rodents, PAG receives input from the medial prefrontal cortex, insular cortex, anterior cingulate cortices, and amygdala (Rizvi et al., 1991; Gabbott et al., 2005), and neurons in the PAG project to respiratory nuclei in the medulla (Sessle, 1981; Huang et al., 2000; Hayward et al., 2004). Intriguingly, there was a cluster of voxels in the anterior cerebellum that showed resting connectivity with the CoA, and this cluster overlaps with an area that was observed to have increased activations during sniffing (Sobel et al., 1998). Thus, CoA could potentially be involved in the generation of odor-valence-induced sniffing modulations, which occur rapidly and which have been proposed to have a subcortical mechanism (Johnson et al., 2003).

The resting connectivity of CoA also included the mediodorsal thalamus (MdTh). MdTh receives direct input from the primary olfactory cortex in rodents and may play a role in olfactory learning, memory (Inokuchi et al., 1993; Courtiol and Wilson, 2015), and attention (Plailly et al., 2008). Novel evidence from humans suggests that functional connectivity between the MdTh and amygdala modulates taste perceptions (Veldhuizen et al., 2020).

The resting networks of MeA and CoA overlapped substantially, possibly reflecting processes that involve both subregions. This overlap is consistent with animal work showing that the MeA and CoA share functional circuits involved in bonding and olfaction (Keller et al., 2004; Meurisse et al., 2009; Keshavarzi et al., 2015). The shared network included medial-frontal cortex, hypothalamus, mammillary bodies, and the globus pallidus. The medial frontal cortex is reciprocally connected to the MeA in rodents and plays a critical role in regulating social and emotional behaviors (Ko, 2017), fear responses (Greenberg et al., 2013; Karalis et al., 2016), and activity in this area correlates *via* the olfactory system with breathing in rodents (Moberly et al., 2018). GABAergic neurons in the MeA project to both the CoA and globus pallidus in a circuit thought to mediate fear motor behaviors (Bian, 2013). Circuitry between the medial frontal cortex, hypothalamus, and amygdala is involved in stress regulation (Diorio et al., 1993; Spencer et al., 2005; Jaferi and Bhatnagar, 2007).

Given the fact that the MeA and CoA are both parts of the human primary olfactory cortex, and are both intertwined with social and emotional circuitry, we speculate that a circuit involving the MeA and CoA could mediate odor-induced emotional motor responses (MeA), and learning/memory of these responses (CoA). These functions could include social olfactory behaviors observed in humans such as odor-induced mood changes (Kadohisa, 2013), innate olfactory bonding behaviors (Varendi and Porter, 2001), and olfactory threat avoidance behaviors (Johnson et al., 2003; Olsson et al., 2014).

### The Periamygdaloid Complex

The resting connectivity map of PAC included areas involved in olfactory cognition and multisensory integration. Resting connectivity with the orbitofrontal cortex was stronger in the PAC than in the MeA or CoA, and resting connectivity with the anterior olfactory nucleus was only present in the PAC. Both the orbitofrontal cortex and anterior olfactory nucleus have been proposed to be involved in odor object coding (Watanabe et al., 2018; Zhou et al., 2019; Aqrabawi and Kim, 2020), and the anterior olfactory nucleus projects heavily back to the olfactory bulb (Rothermel and Wachowiak, 2014). The orbitofrontal cortex is a multisensory area (Price, 2008; Sharma and Bandyopadhyay, 2020), and may be involved in the reward value (Howard et al., 2015) and conscious perception of odors (Li et al., 2010). The PAC also exhibited strong resting connectivity with the temporal pole, which is a multisensory area that may encode information about the abstract conceptual properties of objects (Peelen and Caramazza, 2012). Previous work has shown that the amygdala is functionally connected to the temporal pole (Bach et al., 2011; Fan et al., 2014). Though this area is poorly understood, the temporal pole may be involved in a variety of functions including social and emotional processing, facial recognition, memory, and theory of mind (Kling and Steklis, 1976; Olson et al., 2007). Activity in the temporal pole correlates with odor familiarity (Royet et al., 1999) and activity in both the temporal pole and the amygdala correlate with the valence of olfactory, visual, and auditory stimuli (Royet et al., 2000). Thus olfactory information involved in these judgments may reach the temporal pole *via* the PAC. The PAC also showed resting connectivity with the fusiform gyrus. The fusiform gyrus is involved in facial recognition and object discrimination. Given that PAC receives monosynaptic input from the olfactory bulb, it is possible that resting connectivity between the PAC, the temporal pole, and the fusiform gyrus reflects an olfactory circuit.

In the brainstem, the PAC showed resting state connectivity with a large cluster corresponding to the pontine nuclei. This is a multisensory area that receives visual, auditory, and somatosensory information from cortical and subcortical regions, and links that information to the cerebellum (Glickstein et al., 1985; Schwarz and Thier, 1999), but the fine anatomy of these pathways is poorly defined in humans. In animals, neurons with visual receptive fields can be found in the pontine nuclei (Baker et al., 1976) and others project to the auditory system (Ohlrogge et al., 2001). Activation in the pontine nuclei has been observed in humans during breath-holding (McKay et al., 2008) and CO2 exposure (Pattinson et al., 2009). Thus the PAC may link olfactory information to this multisensory circuitry and may link olfactory information to distinct areas of the brainstem and cerebellum.

Together, all three olfactory subregions of the amygdala showed resting connectivity with a distinct cluster of voxels in the dorsal pons, possibly corresponding to the parabrachial nucleus (Lavezzi et al., 2004). Neurons in the dorsal pons are involved in the regulation of breathing rhythms (Chamberlin and Saper, 1994) and the same area of dorsal pons shows increased BOLD responses during CO2 exposure in humans (Pattinson et al., 2009). Our findings are in line with macaque work showing that the amygdala projects to numerous areas of the brainstem, most of which project back reciprocally (Price and Amaral, 1981). We speculate that the MeA, CoA, and PAC are all well-positioned to mediate rapid, odor-induced changes in respiration and potentially other autonomic functions in response to olfactory input, through these brainstem networks.

## Limitations

One limitation of this study is that the resting state maps we identified do not necessarily reflect networks that carry out olfactory functions. The amygdala is involved in a multitude of processes across sensory systems, and while the fact that a subregion receives input from the olfactory bulb implicates that subregion in olfactory processing, it does not necessitate that its resting network represents exclusively olfactory processing. However, given that the MeA, CoA, and PAC are distinct parts of the primary olfactory cortex, receiving roughly a third of the olfactory bulb output (Allison, 1954), it is likely that some of the resting networks we identified here do relate to olfactory functions. Future experimental work is needed to distinguish between the amygdala’s olfactory and non-olfactory networks. Our hope is that this discussion may lead to new testable hypotheses about these under-studied parts of the primary olfactory cortex.

## Conclusion

The MeA, CoA, and PAC are the only subregions of the amygdala that receive direct input from the olfactory bulb, but little is known about their functions in the human brain. We found that the MeA, CoA, and PAC have distinct resting state networks, and we hypothesize that these networks may underlie distinct olfactory and multisensory processes.

## Data Availability Statement

The raw data supporting the conclusions of this article will be made available by the authors, without undue reservation.

## Ethics Statement

The studies involving human participants were reviewed and approved by Northwestern University’s Institutional Review Board under Protocol #STU00201746. The patients/participants provided their written informed consent to participate in this study.

## Author Contributions

The project was conceived by CZ and TN. TN and GZ carried out the fMRI analyses. TN, GZ, QY, GL, and CZ wrote the manuscript together. All authors contributed to the article and approved the submitted version.

## Conflict of Interest

The authors declare that the research was conducted in the absence of any commercial or financial relationships that could be construed as a potential conflict of interest.

## Publisher’s Note

All claims expressed in this article are solely those of the authors and do not necessarily represent those of their affiliated organizations, or those of the publisher, the editors and the reviewers. Any product that may be evaluated in this article, or claim that may be made by its manufacturer, is not guaranteed or endorsed by the publisher.

## Footnotes

^1^ https://fsl.fmrib.ox.ac.uk/fsl/fslwiki

## Supplementary Materials

Supplementary Figure 1 K-means parcellation of the olfactory amygdala subregions based on resting state connectivity. When k is set to values that do not directly correspond to the number of olfactory subregions, overlap between olfactory amygdala subregions and clusters is reduced. When k is set to 6, the clusters tightly align to the three olfactory amygdala subregions on the left and right sides.

Supplementary Figure 2 Maps of voxels with significant resting state connectivity with olfactory amygdala subregions. In contrast to Figures 4–6, which show areas of distinct connectivity with each subregion, these maps show the complete whole-brain network for each subregion. R, Right hemisphere; L, Left hemisphere; P, Posterior; A, Anterior.
