# Supplementary material for: Human Primary Olfactory Amygdala Subregions Form Distinct Functional Networks, Suggesting Distinct Olfactory Functions
Source: Front Syst Neurosci. 2021 Dec 9;15:752320. doi: 10.3389/fnsys.2021.752320 (PMC8695617; doi:10.3389/fnsys.2021.752320)
Supplement: Supplementary Figure 1 — K-means parcellation of the olfactory amygdala subregions based on resting state connectivity. When k is set to values that do not directly correspond to the number of olfactory subregions, overlap between olfactory amygdala subregions and clusters is reduced. When k is set to 6, the clusters tightly align to the three olfactory amygdala subregions on the left and right sides. [file Data_Sheet_1.pdf]

Supplementary Figures

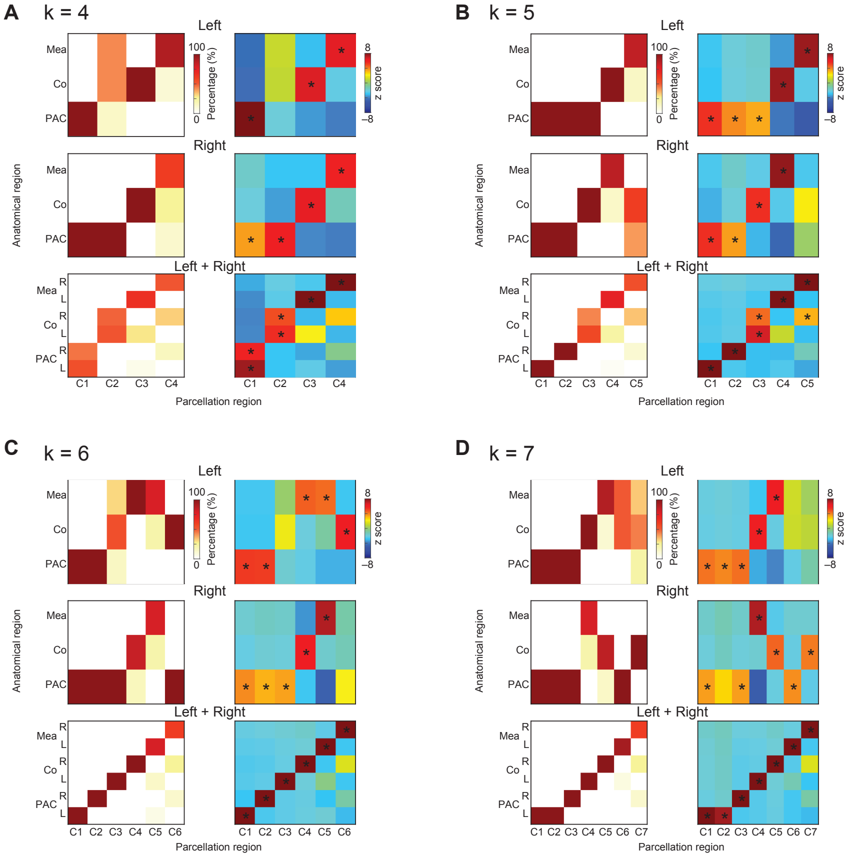

Figure S1. K-means parcellation of the olfactory amygdala subregions based on resting state connectivity. When k is set to values that don't directly correspond to the number of olfactory subregions, overlap between olfactory amygdala subregions and clusters is reduced. When k is set to 6, the clusters tightly align to the three olfactory amygdala subregions in the left and right side.

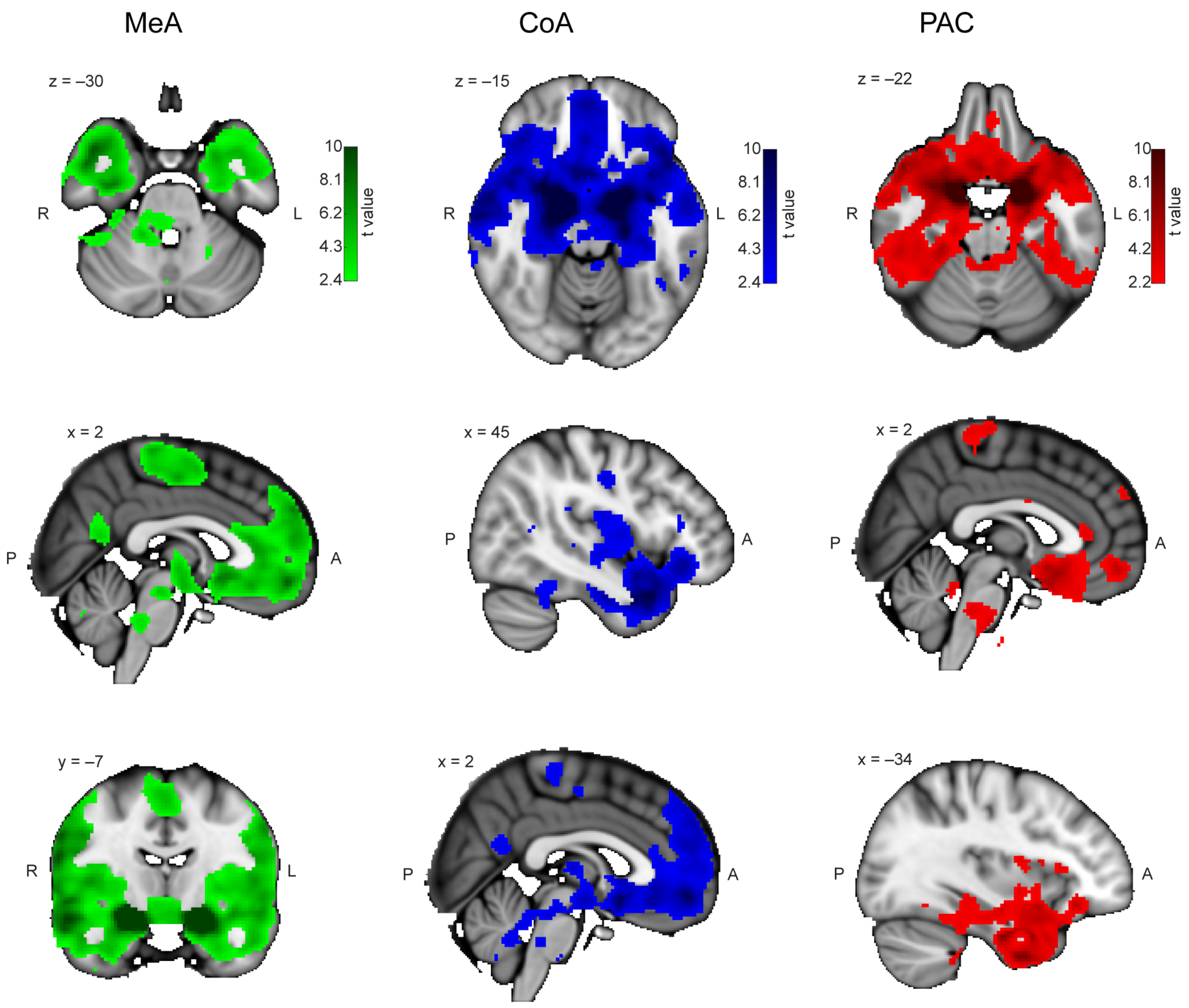

Figure S2: Maps of voxels with significant resting state connectivity with olfactory amygdala subregions. In contrast to figures 4-6, which show areas of distinct connectivity with each subregion, these maps show the complete whole brain network for each subregion. R, Right hemisphere; L, Left hemisphere; P, Posterior; A, Anterior.
